# Valorisation of Barley Straw for Sustainable Nanocellulose Production via Subcritical Alkaline Hydrolysis and HDES-Assisted Processing

**DOI:** 10.3390/molecules31030451

**Published:** 2026-01-28

**Authors:** Dileswar Pradhan, Swarna Jaiswal, Brijesh K. Tiwari, Amit K. Jaiswal

**Affiliations:** 1 Centre for Sustainable Packaging and Bioproducts Research, Technological University Dublin—City Campus, Central Quad, Grangegorman, D07 ADY7 Dublin, Ireland; dileswar.pradhan@tudublin.ie (D.P.); swarna.jaiswal@tudublin.ie (S.J.); 2 School of Food Science and Environmental Health, College of Sciences and Health, Technological University Dublin—City Campus, Central Quad, Grangegorman, D07 ADY7 Dublin, Ireland; 3 Sustainability and Health Research Hub (SHRH), Technological University Dublin—City Campus, Central Quad, Grangegorman, D07 ADY7 Dublin, Ireland; 4 Teagasc Food Research Centre, Ashtown, D15 DY05 Dublin, Ireland; brijesh.tiwari@teagasc.ie

**Keywords:** agricultural waste, subcritical, hydrated deep eutectic solvent, nanocellulose, ultrasound

## Abstract

This study investigates a sustainable and efficient approach to valorising barley straw by producing nanocellulose via an integrated subcritical alkaline hydrolysis and HDES-assisted processing pathway. Subcritical alkaline pretreatment under best processing conditions (150 bar, 200 °C, 125 min) enabled effective biomass fractionation, achieving average hemicellulose and lignin solubilisation of 57.72% and 82.69%, respectively. Subsequent purification of the pretreated solid fraction yielded cellulose fibres with an average cellulose yield of 41.97% and a purity of 87.87%. Nanocellulose was then obtained using a sequential HDES treatment followed by high-intensity ultrasound (HIUS), producing a sample (NC-BTW-3) in which 66% of particles exhibited diameters below 100 nm, 15.2% were between 100 and 200 nm, and 19% were within the 200–1000 nm range. The resulting nanocellulose demonstrated good colloidal stability, with an average zeta potential of −33.0 mV. Overall, the work highlights a green and effective processing strategy for the valorisation of agricultural residues into high-value nanocellulose suitable for bio-based material applications.

## 1. Introduction

Nanocellulose is a naturally occurring nanomaterial derived from cellulose fibres, characterised by at least one dimension measuring less than 100 nanometres [1]. Nanocellulose exists in three primary forms: cellulose nanofibres (CNF), cellulose nanocrystals (CNC), and bacterial nanocellulose (BNC), each possessing unique properties suited for applications in packaging, biomedicine, coatings, and advanced composites. Lignocellulosic biomass (LCB), including wood, agricultural residues, and agro-food industrial waste, serves as a primary feedstock for nanocellulose production [2,3]. The extraction of high-value components, such as cellulose, requires pretreatment steps to break down the complex biomass structure.

Common pretreatment methods include acid and alkali treatments, ultrasonication, microwave irradiation, steam explosion, ionic liquids, and deep eutectic solvents (DES), among others [4]. However, conventional pretreatment methods often suffer from limitations such as high chemical consumption, environmental concerns, and incomplete lignin removal, necessitating the development of more efficient and sustainable approaches. Integrating multiple pretreatment techniques, either simultaneously or sequentially, improves biomass fractionation efficiency and enhances the yield of cellulose and other valuable components [5]. For example, alkali treatment (NaOH) is often coupled with process intensification techniques, such as ultrasound [6,7], microwave irradiation [8,9], high-pressure homogenisation [10], and pulsed electric fields [11], to enhance biomass deconstruction efficiency.

In recent years, the pretreatment of LCB using solvents under high-pressure and high-temperature conditions has emerged as an innovative extraction technique for biomass components. One such method is subcritical water extraction (SWE), where water is pressurised to remain in its liquid state within a temperature range of 100 °C to 374 °C [12]. The SWE technique has been investigated as a pretreatment method for various LCB sources, including sesame hull [12], barley straw [13], and wetland reed grass [14]. Similarly, pressurised aqueous ethanol has been reported as an effective pretreatment method for barley straw [13] and canola straw [15]. These studies highlight the potential of developing efficient pretreatment technologies by exploring organic, acidic, alkaline, and alternative solvents under high-pressure and high-temperature conditions. Advancements in solvent-based pretreatment strategies contribute to improved biomass fractionation efficiency, making them valuable for nanocellulose production and other biorefinery applications.

The development of efficient and sustainable pretreatment techniques capable of producing high-quality cellulose fibres is crucial for enhancing the efficiency of subsequent nanocellulose production processes. In recent years, nanocellulose has been produced from various cellulosic sources using a range of processing technologies, including enzymatic hydrolysis [16], deep eutectic solvents (DES) [17], ionic liquids [18], microfluididation [19], high-intensity ultrasound (HIUS) [20], acid hydrolysis [21], ball milling [22], high-pressure homogenisation [23], and grinding [24], among others. Additionally, several studies have explored hybrid processing methods, where multiple techniques are applied either concurrently or sequentially to enhance the efficiency of nanocellulose extraction [25,26,27,28]. Recently, hydrated deep eutectic solvents (HDES) have been investigated as a pretreatment method for cellulose before nanofibrillation using mechanical techniques [25]. To the best of the authors’ knowledge, the application of a specific HDES formulation comprising betaine, tartaric acid, and water for nanocellulose synthesis, either as a standalone method or in combination with other techniques, has not yet been reported. Furthermore, a notable research gap remains in the pretreatment of biomass using alkaline solvents under subcritical conditions, warranting further investigation.

We hypothesise that combining subcritical alkaline pretreatment with HDES-assisted treatment and HIUS processing will enable the sustainable production of high-purity nanocellulose from barley straw, with improved morphological and functional properties suitable for bio-based material applications. The subcritical alkaline pretreatment is expected to enhance delignification and hemicellulose solubilisation, which are critical steps for obtaining cellulose suitable for nanoscale fibrillation. Further, HDES prepared using betaine, tartaric acid, and water may increase fibre accessibility and promote the loosening of cellulose fibrils, thereby facilitating more efficient nanofibrillation during the subsequent HIUS process.

Given these research gaps and opportunities, this study aimed to achieve three key objectives. First, it sought to develop a pretreatment procedure for barley straw biomass using an alkaline solvent (NaOH) under subcritical conditions to extract cellulose-rich solid materials (CRSM). Second, it aimed to establish a purification method for CRSM to obtain high-purity cellulose fibres (PCF). Finally, the study investigated the conversion of cellulose fibres into nanocellulose through a sequential process involving HDES treatment followed by HIUS treatment.

## 2. Results and Discussion

### 2.1. Subcritical Alkaline Hydrolysis Pretreatment

The composition analysis of barley straw biomass revealed the presence of three key monosaccharides: glucose, xylose, and arabinose. The cellulose content was determined based on the glucose concentration, while the hemicellulose content was calculated as the sum of xylose and arabinose. The barley straw composition was as follows: cellulose (44.09 ± 1.11%), hemicellulose (24.41 ± 0.75%), lignin (19.84 ± 1.28%), ash (4.30 ± 0.15%), and non-lignocellulosic components (7.37 ± 0.87%).

The various pretreatment conditions (PTC) for the subcritical alkaline hydrolysis of barley straw biomass are summarised in Table 1. The ramp-up time required to reach the target pressure and temperature in the SWE system varied depending on the specific PTC, whereas the cooling time was consistently maintained at 20 min across all conditions. After pretreatment, the composition of the solid residue was analysed, and the results are presented in Table 2. The average solid recovery ranged between 56.67% and 70.52%.

Maintaining a constant pressure of either 100 bar or 150 bar and a holding time of either 60 or 90 min, while increasing the temperature from 150 °C to 200 °C, resulted in a decrease in solid recovery, indicating greater solubilisation of biomass components at higher temperatures. Similarly, when maintaining a constant pressure of either 100 bar or 150 bar and a temperature of either 150 °C or 200 °C, while increasing the holding time from 60 to 90 min, a notable reduction in solid recovery was observed. Additionally, when temperature (150 °C or 200 °C) and holding time (60 or 90 min) were kept constant, but pressure was increased from 100 to 150 bar, a slight decrease in solid recovery was recorded; however, this reduction was not statistically significant. These results suggest that holding time and temperature had a significant influence on solid recovery, whereas pressure had a comparatively lower impact. The minimum solid recovery of 56.67 ± 1.25% was observed under PTC-8 conditions (150 bar, 200 °C, 90 min holding time), while the maximum solid recovery of 70.52 ± 1.23% was recorded for PTC-1 (100 bar, 150 °C, 60 min holding time).

The average cellulose content in the pretreated solid residue ranged from 59.89% to 73.21%. Compared to the cellulose content of raw barley straw (44.09%), a significant increase in cellulose content was observed following subcritical alkaline treatment. When pressure was maintained at either 100 or 150 bar and holding time at either 60 or 90 min, increasing the temperature from 150 to 200 °C resulted in a 3% to 4% increase in cellulose content in the pretreated solid residue. However, increasing the pressure from 100 to 150 bar while maintaining a constant holding time of either 60 or 90 min and a constant temperature of either 150 or 200 °C did not lead to a significant change in cellulose content. Huerta et al. [29] similarly reported that varying the pressure from 50 to 200 bar at 180 °C had no significant effect on carbohydrate removal from barley and canola straws after SWE hydrolysis. In contrast, a statistically significant increase in cellulose content was observed when the holding time was extended from 60 to 90 min, while maintaining a constant pressure of either 100 or 150 bar and a temperature of either 150 or 200 °C. The maximum cellulose content of 73.21 ± 2.05% was recorded under PTC-8 conditions (150 bar, 200 °C, 90 min holding time).

The average hemicellulose content in the pretreated solid residue ranged from 15.69% to 18.22%, whereas the initial hemicellulose content of raw barley straw was 24.41%, indicating a significant reduction after pretreatment. Despite variations in pressure, temperature, and holding time, no statistically significant changes in hemicellulose content were observed in the pretreated solid residue. However, the average hemicellulose solubilisation efficiency after pretreatment ranged from 48.92% to 62.70%. A significant increase in hemicellulose solubilisation efficiency was recorded when the temperature was raised from 150 to 200 °C and the holding time extended from 60 to 90 min, while maintaining a pressure of 100 bar. Apart from this, no statistically significant differences were observed with variations in pressure, temperature, and holding time. The maximum hemicellulose solubilisation efficiency of 62.70 ± 1.83% was observed for PTC-6, while PTC-8 resulted in 57.72 ± 0.81% hemicellulose solubilisation efficiency.

The average lignin content in the pretreated solid residue ranged from 6.04% to 17.81%. Depending on the PTC used, the lignin content significantly decreased compared to the initial lignin content of raw barley straw (19.84%). A reduction in lignin content was observed when the temperature increased from 150 to 200 °C, while maintaining a constant pressure of either 100 or 150 bar and a holding time of either 60 or 90 min. However, a statistically significant reduction in lignin content, from 10.37% to 6.04%, was observed only when the temperature was increased from 150 to 200 °C, while maintaining a constant pressure of 150 bar and a holding time of 90 min. Increasing the pressure from 100 to 150 bar, while keeping temperature and holding time constant, resulted in a slight but statistically insignificant reduction in lignin content. Additionally, a significant reduction in lignin content was observed when the holding time increased from 60 to 90 min, while maintaining a constant pressure of either 100 or 150 bar and a temperature of 150 to 200 °C. The minimum lignin content of 6.04 ± 1.20% was recorded under PTC-8 conditions (maximum pressure, maximum temperature, longest holding time).

The average lignin solubilisation efficiency after pretreatment ranged from 36.68% to 82.69%. A statistically significant increase in lignin solubilisation efficiency was observed when the temperature increased from 150 to 200 °C, while maintaining a constant pressure of either 100 or 150 bar and a holding time of either 60 or 90 min. Similarly, an increase in lignin solubilisation efficiency was observed when the pressure was increased from 100 to 150 bar, while maintaining a temperature of either 150 or 200 °C and a holding time of either 60 or 90 min, although the difference was not statistically significant. A significant increase in lignin solubilisation efficiency was also noted when the holding time was extended from 60 to 90 min, while maintaining a constant pressure of either 100 or 150 bar and a temperature of 150 to 200 °C. The maximum lignin solubilisation efficiency of 82.69 ± 3.81% was recorded under PTC-8 conditions (maximum temperature, pressure, and holding time).

During the pretreatment, increasing the temperature from 150 to 200 °C under subcritical alkaline conditions increased cellulose content and decreased solid recovery. The rise in temperature might have accelerated alkali-catalysed reactions, including the cleavage of lignin–carbohydrate complexes (LCCs) and β-O-4 ether bond scission in lignin, as well as hemicellulose depolymerisation, which resulted in a solid residue containing higher cellulose content [29,30]. On the other hand, increasing pressure from 100 to 150 bar (at constant temperature and holding time) did not significantly improve cellulose content because, under subcritical conditions, pressure primarily ensured that the solvent remained in the liquid state, whereas reaction kinetics and solvent aggressiveness are governed mainly by the temperature and alkalinity of the solvent [13]. Further, increasing the holding time from 60 to 90 min increased cellulose content by enabling deeper solvent penetration into fibre walls and the more complete solubilisation of hemicellulose and lignin, including LCCs domains that are highly recalcitrant. A similar trend of time-dependent solubilisation behaviour has been reported in subcritical extraction systems [31].

Several studies have reported the use of high-pressure and high-temperature conditions with other solvents for the biorefinery of similar biomass resources. For instance, Liu et al. [13] reported that pressurised aqueous ethanol (PAE) treatment (60% ethanol) applied to barley straw at 220 °C, 50 bar, and 40 min resulted in the highest cellulose content in the solid residue (88.43%) and the highest lignin removal rate (7.50 ± 0.44% lignin remaining). In another study, Huerta et al. [29] conducted SWE and PAE treatments on barley and canola straw and found that PAE treatment had superior delignification efficiency. Their study reported that hydrolysis with PAE (20% ethanol, 180 °C, 50 bar, 5 mL/min, 40 min) resulted in lignin removal efficiencies of 54% and 45% from barley and canola straw, respectively.

### 2.2. Cellulose Purification and Lignin Recovery

The solid residue obtained from subcritical alkaline hydrolysis pretreatment under PTC-8 conditions was purified to obtain purified cellulose fibres (PCF). The PTC-8 pretreatment condition was selected based on multiple criteria, including minimum solid recovery, lowest lignin content, highest cellulose content, maximum lignin solubilisation, and sufficient hemicellulose solubilisation. The purification process resulted in a PCF yield of 41.97 ± 1.26%, with a cellulose purity of 87.87 ± 1.28%. In a comparable study, Liu et al. [30] extracted cellulose fibres from barley straw using a process consisting of pressurised aqueous ethanol treatment, ultrasound treatment, and alkaline hydrogen peroxide treatment. The extracted cellulose fibres exhibited a maximum purity of 91.1 ± 2.3% [30].

### 2.3. Hydrated DES Treatment of PCF

Pre-treating cellulose fibres with HDES can enhance cellulose accessibility, facilitating ultrasonic energy penetration for the breakdown of cellulose pulp at the nanoscale [25]. Ma et al. [25] demonstrated that HDES combined with ultrasonic treatment effectively produced nanocellulose from kraft pulp. Similarly, Hardiningtyas et al. [32] utilised HDES pretreatment of cellulose fibres isolated from industrial agar seaweed waste biomass, followed by mechanical treatment (sonication and high-speed homogenisation) to produce CNF.

In the present study, PCF was pretreated using different BTW HDES formulations (BTW-1, BTW-2, BTW-3, and BTW-4) under varying process conditions (PCTF) in an autoclave. The PCF recovery after HDES treatment is presented in Table 3. For all four HDES formulations, PCF recovery significantly decreased as the autoclave temperature increased from 100 °C to 120 °C, while the holding time remained constant at either 30, 60, 90, or 120 min. Additionally, PCF recovery decreased (although not always statistically significant) with an increase in treatment time, while maintaining a constant autoclave temperature of either 100 °C or 120 °C.

However, for the same process conditions (PCTF), changing the type of BTW HDES used for treatment did not result in a significant difference in PCF recovery. These findings suggest that treatment temperature and autoclave holding time significantly influence the hydrolysis of PCF, whereas modifications in HDES composition did not have a statistically significant effect on hydrolysis efficiency. The lowest PCF recovery was recorded when the highest temperature (120 °C) and longest holding time (120 min) were applied across all four HDES treatments.

The average PCF recovery across all four HDES treatments ranged between 79% and 95%. To determine the best-performing process condition (PCTF) for further downstream processing, the PCF recovery target was set at approximately 85%, ensuring that PCF hydrolysis was neither excessive nor insufficient. Based on these criteria, PCTF-6 was selected as the best process condition for further processing. The PCTF-6 parameters included an autoclave temperature of 120 °C, a holding time of 60 min, a ramp-up time of 25 min, and a cooling time of 25 min. Under these conditions, the average PCF recovery for BTW-1, BTW-2, BTW-3, and BTW-4 was 84.93%, 84.30%, 82.98%, and 82.52%, respectively.

### 2.4. Characterisation

#### 2.4.1. Dynamic Light Scattering (DLS) Analysis

**Figure 1:**
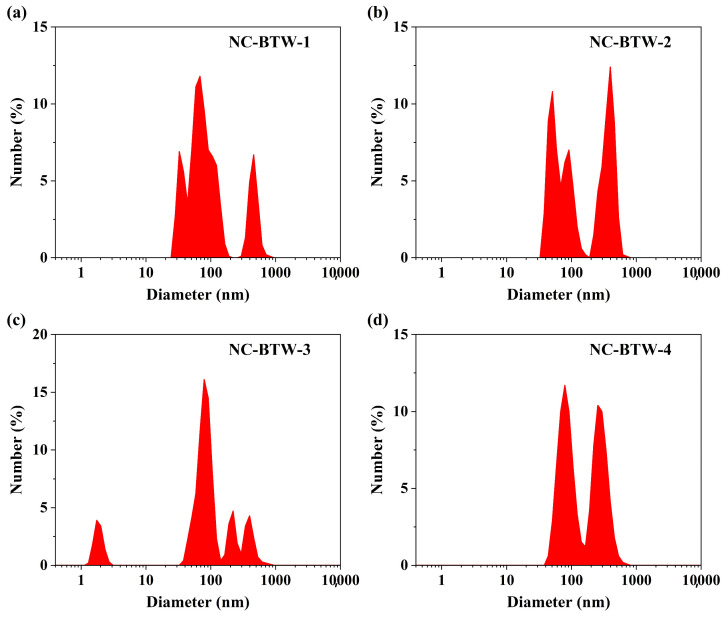
Number size distribution plots of NC-BTW nanocellulose samples; (**a**) NC-BTW-1; (**b**) NC-BTW-2; (**c**) NC-BTW-3; (**d**) NC-BTW-4.

The particle size distribution, polydispersity index (PDI), and zeta potential of the four nanocellulose samples were evaluated using DLS analysis. The number-based particle size distribution plots for NC-BTW-1, NC-BTW-2, NC-BTW-3, and NC-BTW-4 are shown in Figure 1a, Figure 1b, Figure 1c and Figure 1d, respectively.

For NC-BTW-1, approximately 65.2% of nanoparticles had a diameter of less than 100 nm, 16.9% had a diameter between 100 and 200 nm, and 17.8% had a diameter between 200 and 1000 nm. In the case of NC-BTW-2, 47.4% of nanoparticles had a diameter of less than 100 nm, 7.4% had a diameter between 100 and 200 nm, and 44.7% fell within the 200–1000 nm range. For NC-BTW-3, 66.0% of nanoparticles had a diameter of less than 100 nm, 15.2% were between 100 and 200 nm, and 19.0% were between 200 and 1000 nm. In contrast, NC-BTW-4 had the lowest proportion of smaller particles, with 41.7% having a diameter of less than 100 nm, 16.0% between 100 and 200 nm, and 34.5% between 200 and 1000 nm.

Tuning the composition of the BTW HDES might have influenced various characteristics, such as hydrogen-bond acidity, hydrogen-bond basicity, and overall solvating behaviour, thereby affecting the extent of cellulose fibril loosening during HDES treatment. In some studies, it has been demonstrated that DES with higher acidity provide more active protons and induce stronger disruption of the native hydrogen-bond network within cellulose, thereby enhancing fibre swelling and loosening before mechanical nanofibrillation [25,33]. In this study, similar results were observed for the BTW-3 HDES formulation, which contained a higher proportion of tartaric acid. The BTW-3 HDES-treated cellulose was utilised for production of NC-BTW-3 nanocellulose, which had highest proportion of nanoparticles below 100 nm following HIUS treatment. In contrast, the other BTW HDES formulations, which contained lower tartaric acid levels, may have led to reduced fibre disintegration efficiency, thereby yielding broader particle size distributions and a larger fraction of particles above 200 nm.

Several studies have reported DLS-based particle size distribution analyses for nanocellulose derived from similar biomass sources. Gond et al. [34] isolated cellulose nanofibres (CNF) from sugarcane bagasse using sodium bicarbonate treatment followed by mechanical grinding. Their nanocellulose particle size distribution ranged from 56.70 nm to 221.90 nm, with approximately 55% of the particles measuring around 75 nm [34]. Similarly, Trivedi et al. [35] extracted nanocellulose from wheat straw using a combination of chemical treatments followed by grinding and sieving, reporting particle sizes between 13 and 50 nm, with approximately 90% of nanoparticles having a size of 30 nm [35]. In another study, Al-haql et al. [36] isolated spherical nanocellulose from sesame husks using a series of chemical treatments followed by ultrasonication. They reported that the nanocellulose had an average diameter of 108.1 nm and exhibited a bimodal distribution with two peaks at diameters of 25.02 nm and 138.6 nm [36].

The PDI measurement of a nanoparticle suspension indicates the width of the particle size distribution. A PDI value close to 1 signifies a highly polydisperse system, while a PDI value close to 0 indicates a highly monodisperse sample [37]. The PDI values for NC-BTW-1, NC-BTW-2, NC-BTW-3, and NC-BTW-4 were 0.883 ± 0.134, 0.695 ± 0.11, 0.812 ± 0.097, and 0.705 ± 0.122, respectively. No statistically significant differences were observed among the four nanocellulose samples in terms of PDI values.

The average zeta potential of all four nanocellulose samples was lower than −30 mV, indicating a stable dispersion of nanocellulose in aqueous medium. The zeta potential values for NC-BTW-1, NC-BTW-2, NC-BTW-3, and NC-BTW-4 were −31.5 ± 4.27 mV, −35.9 ± 2.78 mV, −33.0 ± 3.6 mV, and −32.6 ± 3.43 mV, respectively. No statistically significant differences were observed among the four nanocellulose samples regarding zeta potential.

Comparable zeta potential values have been reported for nanocellulose extracted from similar biomass sources, such as mustard straw (−25.98 mV) [38], wheat bran (−36.5 mV to −39.8 mV) [39], rice husks (−21.3 mV to −33.8 mV) [40], soybean straw (−24.5 mV for CNF and −28.8 mV for CNC) [41], sesame husks (−27.2 mV) [36], and rice straw (−42.0 mV) [42].

Considering the high proportion of nanoparticles smaller than 100 nm, along with the low polydispersity index (PDI) and stable zeta potential, the NC-BTW-3 sample was selected as the best sample for nanocellulose preparation using a sequential process comprising HDES treatment followed by HIUS treatment. An image of the NC-BTW-3 nanocellulose sample is shown in Figure S1. The NC-BTW-3 sample was further characterised using Fourier transform infrared spectroscopy (FTIR) and X-ray diffraction (XRD) analysis.

#### 2.4.2. FTIR

**Figure 2:**
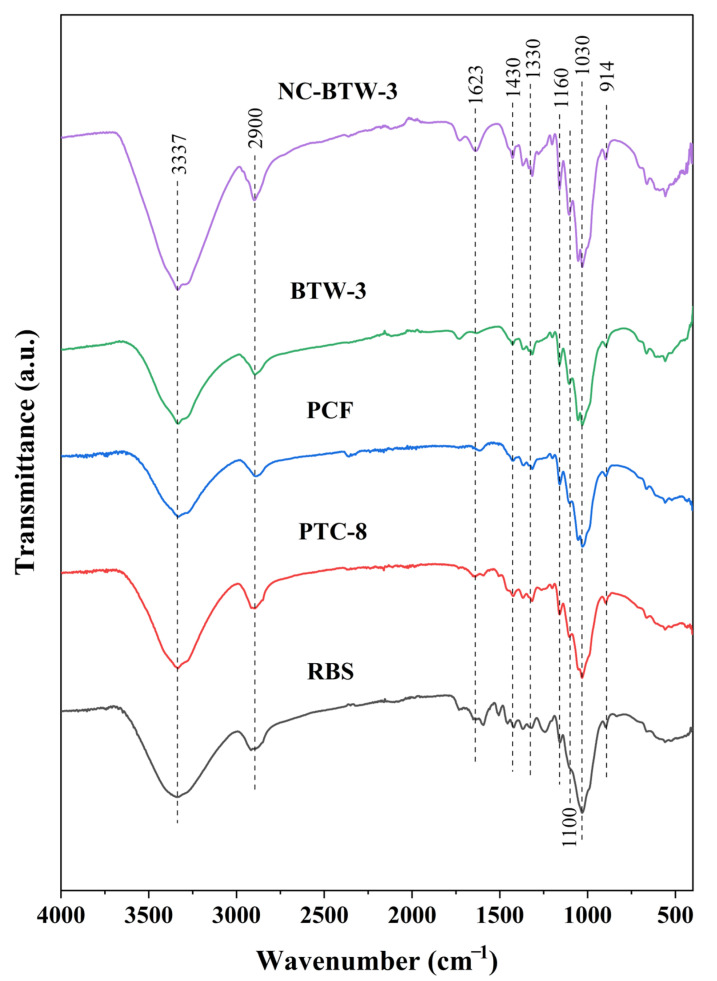
FTIR spectra of RBS, PTC-8, PCF, BTW-3 (HDES-treated cellulose), and NC-BTW-3 samples.

The FTIR spectra of raw barley straw (RBS), PTC-8, PCF, BTW-3, and NC-BTW-3 samples are shown in Figure 2. A broad absorption band observed in the 3000–3600 cm^−1^ region corresponds to O–H stretching and bending vibrations, which are associated with hydrogen-bonded hydroxyl (OH) groups of cellulose and absorbed water [43]. A peak in the 2900 cm^−1^ region was detected in all samples and is attributed to the symmetrical and asymmetrical stretching vibrations of C–H bonds in cellulose, confirming the preservation of the cellulose backbone throughout the process [44].

The peak at 1623 cm^−1^ is assigned to chemically adsorbed water and its bending angular deformation [45]. The peak at 1623 cm^−1^ showed the highest intensity in the spectra of the NC-BTW-3 nanocellulose sample; however, the peak was almost non-existent in other samples. The higher intensity of this peak in the NC-BTW-3 spectrum might be due to the greater specific surface area and higher density of surface hydroxyl sites available in nanocellulose sample, which favours interfacial water adsorption and retention at nanofibril surfaces [46].

The peak at 1430 cm^−1^ corresponds to CH_2_ bending, linked to intermolecular hydrogen bonding at the C6 group. This peak is related to crystalline cellulose vibrations and becomes more prominent after purification, consistent with increased cellulose ordering and the removal of amorphous components [43]. This observation is consistent with the increase in the crystallinity index observed throughout the nanocellulose production process, as confirmed by the XRD analysis. Peaks detected in the 1369–1330 cm^−1^ range are associated with C–H and C–O groups of polysaccharides [47].

The peaks observed around 1100 cm^−1^ and 1160 cm^−1^ correspond to C–C ring bending vibrations and the C–O–C glycosidic ether bond of the β-1,4 glycosidic linkage between D-glucose units in cellulose [44]. The increased sharpness and intensity of these peaks in BTW-3 and NC-BTW-3 confirmed the retention of the cellulose I crystalline structure after HDES treatment and HIUS processing [44]. Additionally, the peaks at 1030 cm^−1^ and 914 cm^−1^ are attributed to C–O stretching and C–H deformation vibrations, which are characteristic of cellulose structure [48].

#### 2.4.3. XRD

The supramolecular structure of cellulose consists of a hierarchical organisation of crystalline and amorphous domains, governed by the spatial arrangement of cellulose chains, hydrogen-bonding networks, and microfibrillar packing [49,50]. In a study, Makarov et al. [51] described that cellulose fibrils consist of highly ordered crystalline regions embedded within less ordered domains (amorphous portion), producing a multilevel structural arrangement that directly influences the mechanical, thermal and other characteristics of the material [51]. The sequential processing of barley straw through subcritical alkaline hydrolysis, purification, HDES treatment, and HIUS nanofibrillation progressively removed lignin and hemicellulose, thereby exposing and reorganising the hierarchical supramolecular structure of cellulose. This restructuring increased the overall crystallinity and reduced structural disorder, which was determined by XRD analysis.

**Figure 3:**
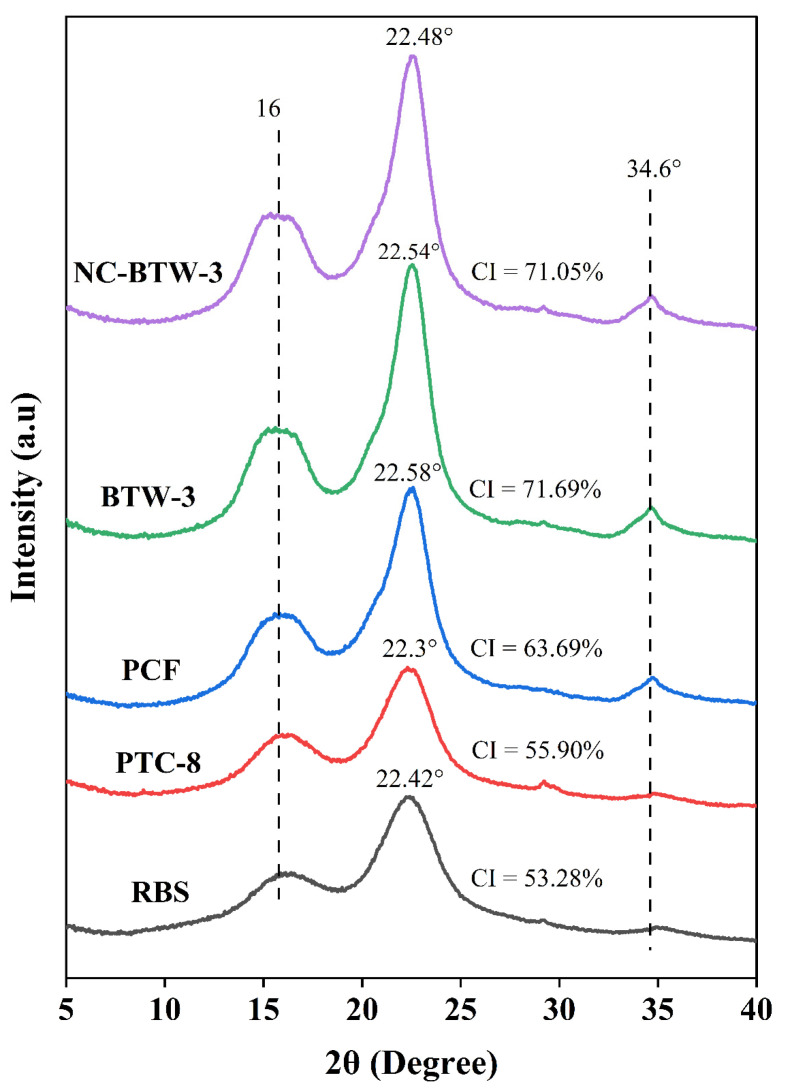
XRD analysis of RBS, PTC-8, PCF, BTW-3 (HDES-treated cellulose) and NC-BTW-3 samples.

The crystallinity properties of the biomass samples at various stages of nanocellulose production were evaluated using X-ray diffraction (XRD) analysis. The XRD patterns of raw barley straw (RBS), PTC-8, PCF, BTW-3, and NC-BTW-3 are presented in Figure 3. In the low-angle region (2θ between 14 and 16°), two partially overlapping peaks were observed at approximately 14° and 15.5°, which confirmed the presence of cellulose I structure. The peaks detected near a 2θ angle of 16° correspond to the (110) crystallographic planes, which are characteristic of the cellulose I structure [52,53]. Additionally, the peak observed around 34.6° corresponds to the (004) crystalline planes, further confirming the presence of cellulose I [54]. The sharp and well-defined peak at 34.6° in PCF, BTW-3, and NC-BTW-3 suggests a higher purity of cellulose in these samples. Prominent peaks were also observed in all samples between 22.3° and 22.58°, corresponding to the 002 plane, which is attributed to cellulose I [55]. The intensity of peaks between 22° and 24° increased progressively after each treatment step, indicating enhanced refinement and structural organisation of the biomass as it was converted into nanocellulose. The XRD findings confirm that the native cellulose I structure remained intact throughout the nanocellulose production process from barley straw biomass, indicating that the pretreatment and processing methods did not alter the fundamental crystalline structure of cellulose.

The CI of all samples was calculated using Segal’s method. The CI values for raw barley straw (RBS), PTC-8, PCF, BTW-3, and NC-BTW-3 were determined to be 53.28%, 55.90%, 63.69%, 71.69%, and 71.05%, respectively. A significant increase in CI was observed after each treatment step, which could be attributed to the removal of lignin, hemicellulose, and other amorphous non-cellulosic components [53]. Additionally, no significant difference was observed between the CI values of BTW-3 and NC-BTW-3, indicating that the final HIUS treatment for nanocellulose production did not alter the crystallinity properties of the HDES-treated cellulose (BTW-3). The FTIR results further supported this trend, as the sharpening and increased intensity of the cellulose-specific band at 1430 cm^−1^ during nanocellulose production indicated an improvement om crystalline domains.

The CI of nanocellulose (NC-BTW-3) obtained in this study (71.05%) was higher than the CI of nanocellulose derived from soybean straw, where CNF produced via enzymatic treatment had a CI of 50%, and CNC produced via acid hydrolysis had a CI of 57% [41]. Nehra et al. [56] synthesised nanocellulose from wheat straw using a combination of chemical and mechanical treatments and reported a CI of 76.43%, which was comparable to NC-BTW-3 in the current study. Similarly, Xu et al. [57] reported a CI of 76.99% for nanocrystalline cellulose derived from rice straw, using a process that involved chemical treatments followed by ultrasonication. These findings suggest that the CI of nanocellulose is influenced by multiple factors, including the biomass source and the production method employed.

#### 2.4.4. Scanning Electron Microscopy (SEM)

**Figure 4:**
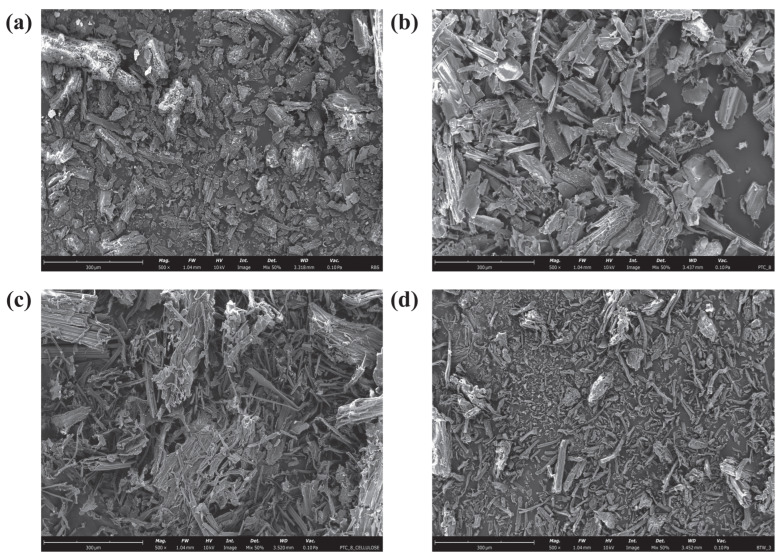
SEM images of (**a**) RBS; (**b**) PTC-8; (**c**) PCF and (**d**) HDES-treated cellulose (BTW-3) samples at a magnification of 500×; Scale bar = 300 μm for all images.

The morphological changes in the biomass samples at different stages of nanocellulose production were examined using scanning electron microscopy (SEM) analysis. The SEM images of raw barley straw (RBS), PTC-8, PCF, and BTW-3 are presented in Figure 4. The SEM image of RBS (Figure 4a) revealed a rough, irregular surface texture with no visible cellulose microfibril structure. This is due to the complex matrix of lignocellulosic components, where cellulose, hemicellulose, and lignin are strongly bonded together. Additionally, non-lignocellulosic compounds are also integrated within this structure, further contributing to the dense and compact morphology.

Following subcritical alkaline hydrolysis pretreatment (PTC-8), the SEM image (Figure 4b) showed a smoother surface structure with distinct visibility of lignocellulosic fibrils, indicating partial removal of amorphous components and disruption of the lignin-hemicellulose network.

After purification (PCF sample, Figure 4c), the SEM image exhibited the presence of both individual cellulose fibrils and fibrillar bundles, demonstrating further removal of non-cellulosic components and partial disintegration of the cellulose matrix. Following HDES treatment (BTW-3, Figure 4d), the SEM image revealed well-separated individual cellulose fibrils with a cylindrical and rod-like morphology. This suggests that HDES effectively facilitated the breakdown of fibril aggregates, leading to enhanced fibrillation and the isolation of cellulose fibres.

## 3. Materials and Methods

### 3.1. Materials Procurement

Barley straw was sourced from a commercial supplier in Dublin, Ireland. The straw was pre-packaged and used as received. It was then ground using a blender, and the ground sample was stored in zip-lock bags for further use. The sugar standards, such as D-(+)-Glucose (CAS: 50-99-7), D-(+)-Xylose (CAS: 58-86-6), D-(+)-Mannose (CAS: 3458-28-4), D-(+)-Galactose (CAS: 59-23-4), and L-(+)-Arabinose (CAS: 5328-37-0), were purchased from Merck, Cork, Ireland. Additionally, other chemicals used in this study, including sulphuric acid (CAS: 7664-93-9), calcium carbonate (CAS: 471-34-1), sodium hydroxide (CAS: 1310-73-2), and sodium hypochlorite (CAS: 7681-52-9), were procured from Merck, Ireland.

### 3.2. Biomass Composition Analysis

The biomass composition analysis was conducted following the laboratory analytical protocol established by the National Renewable Energy Laboratory (NREL) to determine structural carbohydrates and lignin in biomass [58]. The biomass sample was subjected to acid hydrolysis using 72% sulphuric acid at 30 °C for one hour in a water bath. After hydrolysis, the sample was diluted to a 4% acid concentration and autoclaved at 121 °C for one hour. The mixture was then cooled and vacuum-filtered using a filter crucible. The hydrolysis liquor was used to quantify the monosaccharide content and acid-insoluble lignin (AIL), while the acid-insoluble residue (AIR) retained in the filtration crucible was analysed to determine the acid-soluble lignin (ASL) content.

For the determination of ASL, an appropriate amount of hydrolysis liquor was diluted with ultrapure water (dilution factor of 20), and the absorbance of the diluted solution was measured at 280 nm using a UV–visible spectrophotometer. An absorptivity value of 84.8 L g^−1^ cm^−1^ and a path length of 1 cm were used to calculate the ASL content. For the determination of AIL, the AIR content was measured after placing the filter crucible in an oven at 105 °C for 24 h, followed by ashing in a muffle furnace at 575 °C for 24 h to determine the residual ash content. The AIL content was calculated based on the amount of AIR and ash obtained from this process, with the total lignin content reported as the sum of AIL and ASL.

To quantify the monosaccharides, a 10 mL sample of the hydrolysis liquor was transferred into a 50 mL centrifuge tube, and its pH was adjusted to a range of 5 to 6 using calcium carbonate. The mixture was then centrifuged, and the supernatant was filtered through a 0.2 µm syringe filter before being transferred to an HPLC sample vial. Monosaccharide quantification was performed using an Ultra-High-Performance Liquid Chromatography (UHPLC) system equipped with a refractive index detector (RID) and a Biorad Aminex HPX-87P column (Bio-Rad, Hercules, CA, USA). The mobile phase consisted of ultrapure water (0.2 µm filtered and degassed), with the following method parameters: a flow rate of 0.6 mL/min, column temperature of 80 °C, RID temperature of 35 °C, injection volume of 10 µL, and a run time of 20 min. The glucose content in the sample was reported as cellulose content, while the sum of xylose, arabinose, galactose, and mannose was reported as hemicellulose content.

### 3.3. Subcritical Alkaline Hydrolysis Pretreatment

An SWE system operating in batch mode was used for the pretreatment of biomass. Based on preliminary experiments, the alkaline hydrolysis of biomass under subcritical conditions was carried out using 0.5 M NaOH. In all pretreatment experiments, 25 g of biomass was placed into a 500 mL subcritical vessel, and NaOH solution was pumped into the vessel to maintain a solid-to-liquid ratio of 1:20 *w*/*v*. The specific pretreatment conditions (PTC) used for the biomass treatment are provided in Table 1.

Once the designated holding time was reached according to the specified PTC, cold water was pumped into the SWE system in continuous flow mode to halt the reaction and cool the vessel. The cooling water was pumped at a flow rate of 25 mL/min for 20 min. After cooling, the liquid fraction was collected from the collector chamber of the SWE system. The SWE vessel was then opened, and the treated biomass sample was removed.

The liquid and solid fractions were separated by centrifugation (4000× *g* for 15 min). The solid residue obtained after centrifugation was washed twice with distilled water and then freeze-dried for composition analysis and determination of solid recovery rate. This solid residue was designated as cellulose-rich solid material (CRSM). The wet CRSM was subsequently subjected to a purification process to obtain high-purity cellulose fibres. Additionally, pretreatment efficiency parameters, including lignin solubilisation and hemicellulose solubilisation, were determined to evaluate the effectiveness of the process.

### 3.4. Purification of Cellulose

The cellulose-rich solid material (CRSM) obtained from the pretreatment process was purified using a 2% sodium hypochlorite solution [59,60]. The CRSM was mixed with the solvent at a solid-to-liquid ratio of 1:20 *w*/*v* and treated at 60 °C in a water bath for 30 min. After the treatment, the solid and liquid fractions were separated by centrifugation at 4000× *g* for 10 min. The solid fraction, designated purified cellulose fibres (PCF), was washed three times with deionised water and then freeze-dried to determine its yield and purity. The purity of PCF was evaluated using the monosaccharide quantification method (as per Section 3.2).

### 3.5. Production of Nanocellulose

#### 3.5.1. HDES Preparation

The HDES was prepared by combining betaine, tartaric acid, and water at varying molar ratios. The sample codes and compositions of each HDES formulation are provided in Table 4. The appropriate quantities of each component were weighed, mixed in a reaction beaker, and heated on a hot plate at 50 °C with continuous stirring at 300 rpm. The formation of DES was completed in less than 10 min. Once a clear, transparent solvent was formed, it was transferred to glass storage bottles for further use.

#### 3.5.2. HDES Treatment

The HDES treatment was conducted in an autoclave under varying temperature (100 and 120 °C), ramp-up time (20 and 25 min), holding time (30, 60, 90, and 120 min), and cooling time (20 and 25 min) conditions. The ramp-up and cooling times differed depending on the selected temperature. For each experiment, 2 g of purified cellulose fibres (PCF) was mixed with 50 mL of HDES in an autoclave-safe glass bottle, maintaining a solid-to-liquid ratio of 1:25 *w*/*v*. This ratio was kept constant across all experiments, as determined from preliminary trials. Upon completion of the treatment, the PCF residue and liquid fraction were separated by centrifugation (4000× *g* for 10 min). The PCF residue was then washed twice with water to remove any residual HDES. The PCF recovery following BTW HDES treatment was determined, with the recovery efficiency evaluated by freeze-drying the PCF residue. However, the wet PCF residue was further utilised for nanofibrillation using HIUS treatment.

#### 3.5.3. High Intensity Ultrasound Treatment

Based on the PCF recovery following HDES treatment, an appropriate amount of deionised water was added to the PCF sample to maintain a solid concentration of 0.1 wt.% in the PCF–water mixture. The aqueous PCF mixture was stirred at 300 rpm for 5 min before being transferred to an ultrasonic beaker, which was connected to a chiller set at 1 °C. The HIUS treatment was performed for 20 min using a 2 cm diameter ultrasound probe (1000 hdT, 20 kHz, Hielscher, Teltow, Germany) at an output power of 500 W.

### 3.6. Characterisation

#### 3.6.1. Dynamic Light Scattering Analysis

Dynamic light scattering (DLS) analysis was performed using the Zetasizer Nano ZS (Malvern Panalytical, Westborough, MA, USA) to determine the particle size, polydispersity index (PDI), and zeta potential of the sample. The sample was diluted tenfold, and measurements were conducted at a controlled temperature of 25 °C with a scattering angle of 173°.

#### 3.6.2. Fourier Transform Infrared Spectroscopy

The chemical structure of raw biomass, pretreated biomass, cellulose, and nanocellulose samples was analysed using an attenuated total reflectance-Fourier transform infrared (ATR-FTIR) spectrometer (Nicolet iS5, Thermo Scientific, Waltham, MA, USA). The analysis was conducted by performing 64 scans with a resolution of 4 cm^−1^ over a spectral range of 4000 to 400 cm^−1^ in transmittance mode.

#### 3.6.3. Scanning Electron Microscopy

The morphology of biomass samples at different stages of nanocellulose production was examined using a desktop scanning electron microscope (SEM) (Phenom™ XL, Thermo Scientific, Waltham, MA, USA), operating at an accelerating voltage of 10 kV. The powdered samples were mounted on carbon tabs, and SEM images were acquired at a magnification of 500×.

#### 3.6.4. X-Ray Diffraction

The crystallinity properties of the samples were analysed using a Rigaku Miniflex benchtop X-ray diffractometer (Tokyo, Japan), equipped with a monochromatic CuKα radiation source (λ = 1.54059 Å, 40 kV, 15 mA). The scanning parameters included a 2θ angle range of 5° to 40°, a step width of 0.02°, and a scan speed of 2°/min. The crystallinity index (CI) was calculated using Segal’s method as per Equation (1) [61]:

(1) $$CI\left(\%\right)=\frac{I_{002}-I_{am}}{I_{002}}\times 100$$

where I_002_ represents the maximum intensity (in arbitrary units) of the 002-lattice diffraction at a 2θ angle between 22° and 24° [55], and Iam is the minimum diffraction intensity in the same units at a 2θ angle between 18° and 19° [62]. I_002_ accounts for both amorphous and crystalline regions, whereas Iam corresponds only to the amorphous phase [63].

### 3.7. Statistical Analysis

All experiments were conducted in triplicate unless otherwise stated, and results are presented as mean ± standard deviation. Statistical analyses were performed using SPSS software (Version 29, IBM, Armonk, NY, USA), including one-way ANOVA and Tukey’s post hoc test. A statistically significant difference was attributed to *p*-values < 0.05.

## 4. Conclusions

This study successfully isolated nanocellulose from barley straw through a sequential processing approach involving subcritical alkaline hydrolysis pretreatment, purification, HDES treatment, and HIUS treatment. The best-performing pretreatment condition (PTC-8) among those tested resulted in an average lignin solubilisation of 82.69% and hemicellulose solubilisation of 57.72%. Further purification of pretreated biomass resulted in high-purity cellulose with a yield of 41.97% and purity of 87.87%. Further, HDES treatment of high-purity cellulose resulted in the effective separation of individual cellulose fibrils, producing well-defined cylindrical and rod-shaped fibres, as confirmed from SEM analysis. The HIUS treatment of HDES-treated cellulose resulted in a high proportion (~66%) of nanoparticles with diameters below 100 nm (NC-BTW-3 sample), as confirmed by DLS analysis. The XRD findings established that the native cellulose I structure remained unchanged, and the crystallinity index increased from 53.28% (raw barley straw) to 71.05% (NC-BTW-3 sample) during nanocellulose production. This study provides a sustainable and efficient approach to nanocellulose isolation from agricultural waste, offering potential applications in biopolymer development, bio-based packaging, and nanocomposite materials. However, despite the promising lab-scale performance, various challenges remain in scaling up the developed process. Subcritical operation at 150 to 200 °C and 100 to 150 bar requires large, high-pressure reactors, high-capacity pumps, and robust heat integration strategies for pretreatment at a large scale. This requires significant capital investment and maintenance cost. In addition, process reliability depends on quality control at all stages, high-throughput solid–liquid separation, and effluent conditioning for alkaline liquors. Further research is required at the pilot scale to address these technical and operational constraints.

## Figures and Tables

**Table 1:** Pretreatment conditions (PTC) of barley straw using subcritical aqueous alkaline extraction (0.5 M NaOH and 1:20 *w*/*v* solid to liquid ratio).

Sample Code	Pressure (bar)	Temperature (°C)	Ramp-Up Time (min)	Holding Time (min)	Cooling Time (min)	Total Processing Time (min)
PTC-1	100	150	10	60	20	90
PTC-2	100	200	15	60	20	95
PTC-3	150	150	10	60	20	90
PTC-4	150	200	15	60	20	95
PTC-5	100	150	10	90	20	120
PTC-6	100	200	15	90	20	125
PTC-7	150	150	10	90	20	120
PTC-8	150	200	15	90	20	125

**Table 2:** Composition of raw and pretreated barley straw at various pretreatment conditions (PTC).

Sample Code	Solid Recovery (%)	Cellulose (%)	Hemicellulose (%)	Lignin (%)	Hemicellulose Solubilisation (%)	Lignin Solubilisation (%)
Raw barley straw	-	44.09 ± 1.11	24.41 ± 0.75	19.84 ± 1.28	-	-
PTC-1	70.52 ± 1.23 ^d^	59.89 ± 1.36 ^a^	17.66 ± 1.75 ^a^	17.81 ± 1.08 ^e^	48.92 ± 5.77 ^a^	36.68 ± 4.10 ^a^
PTC-2	64.31 ± 1.89 ^bc^	64.93 ± 1.41 ^ab^	16.47 ± 1.80 ^a^	15.20 ± 1.12 ^cde^	56.53 ± 6.01 ^ab^	50.80 ± 2.20 ^bc^
PTC-3	68.23 ± 2.35 ^cd^	61.68 ± 2.36 ^a^	16.42 ± 1.17 ^a^	16.82 ± 1.18 ^de^	54.12 ± 3.36 ^ab^	42.15 ± 4.46 ^ab^
PTC-4	63.69 ± 2.63 ^bc^	65.46 ± 2.11 ^abc^	17.22 ± 1.19 ^a^	14.16 ± 1.25 ^cd^	55.01 ± 4.78 ^ab^	54.44 ± 5.86 ^cd^
PTC-5	61.14 ± 1.62 ^ab^	69.10 ± 1.95 ^bcd^	16.82 ± 1.86 ^a^	11.93 ± 1.37 ^bc^	57.96 ± 3.50 ^ab^	63.20 ± 4.85 ^de^
PTC-6	58.06 ± 1.40 ^b^	71.34 ± 2.22 ^d^	15.69 ± 1.04 ^a^	8.83 ± 1.05 ^ab^	62.70 ± 1.83 ^b^	74.13 ± 3.55 ^ef^
PTC-7	59.80 ± 1.45 ^ab^	70. 89 ± 2.36 ^cd^	17.64 ± 1.85 ^a^	10.37 ± 0.97 ^b^	56.72 ± 5.55 ^ab^	68.72 ± 3.44 ^e^
PTC-8	56.67 ± 1.25 ^a^	73.21 ± 2.05 ^d^	18.22 ± 0.75 ^a^	6.04 ± 1.20 ^a^	57.72 ± 0.81 ^ab^	82.69 ± 3.81 ^f^

Different letters in the same column indicate statistically significant differences (*p* < 0.05).

**Table 3:** The recovery of PCF after treatment with four different BTW HDES at various PCF treatment factors (PCTF).

PCF Treatment Factors	Temperature (°C)	Treatment Time in Autoclave (min)			PCF Recovery After HDES Treatment (%)			
		Ramp-Up Time	Holding Time	Cooling Time	BTW-1	BTW-2	BTW-3	BTW-4
PCTF-1	100	20	30	20	94.69 ± 0.79 ^f^	93.72 ± 1.12 ^f^	93.68 ± 1.11 ^f^	91.31 ± 1.57 ^d^
PCTF-2	100	20	60	20	89.88 ± 0.96 ^de^	89.25 ± 1.10 ^de^	89.29 ± 1.02 ^e^	88.42 ± 1.01 ^d^
PCTF-3	100	20	90	20	87.54 ± 0.71 ^cd^	86.92 ± 0.68 ^cd^	87.34 ± 0.63 ^cd^	85.31 ± 1.16 ^c^
PCTF-4	100	20	120	20	85.73 ± 1.16 ^bc^	85.98 ± 0.73 ^bc^	84.96 ± 0.69 ^bc^	84.16 ± 1.05 ^c^
PCTF-5	120	25	30	25	92.57 ± 1.15 ^ef^	91.07 ± 1.39 ^ef^	89.53 ± 0.95 ^e^	88.70 ± 1.05 ^d^
PCTF-6	120	25	60	25	84.93 ± 1.05 ^bc^	84.30 ± 1.28 ^bc^	82.98 ± 1.01 ^ab^	82.52 ± 0.87 ^bc^
PCTF-7	120	25	90	25	83.60 ± 0.75 ^b^	83.81 ± 1.21 ^ab^	82.07 ± 1.29 ^a^	80.36 ± 0.89 ^ab^
PCTF-8	120	25	120	25	80.57 ± 1.34 ^a^	81.16 ± 0.73 ^a^	81.06 ± 1.05 ^a^	79.14 ± 0.52 ^a^

Different letters in the same column indicate statistically significant differences (*p* < 0.05).

**Table 4:** Molar ratios of three components of BTW HDES.

HDES Code	Molar Ratios of Three Components of HDES (B:T:W)		
	Betaine (B) (MW: 117.148 g/mol)	L-(+)-Tartaric Acid (T) (MW: 150.09 g/mol)	Water (W) (MW: 18.01 g/mol)
BTW-1	0.5	1	20
BTW-2	1	1	20
BTW-3	1	1.5	20
BTW-4	1.5	1.5	20

## Data Availability

The datasets generated and/or analysed during the current study are available from the corresponding author upon reasonable request.

## Supplementary Materials

The following supporting information can be downloaded at: https://www.mdpi.com/article/10.3390/molecules31030451/s1, Figure S1. Image of the NC-BTW-3 nanocellulose sample.

## Abbreviations

The following abbreviations are used in this manuscript:

NC	Nanocellulose
CNF	Cellulose nanofibres
CNC	Cellulose nanocrystals
LCB	Lignocellulosic biomass
DES	Deep eutectic solvent
HDES	Hydrated deep eutectic solvent
SWE	Subcritical water extraction
HIUS	High-intensity ultrasound
CRSM	Cellulose-rich solid material
PCF	Purified cellulose fibres
PTC	Pretreatment conditions
DLS	Dynamic light scattering
