# Supplementary material for: Valorisation of Barley Straw for Sustainable Nanocellulose Production via Subcritical Alkaline Hydrolysis and HDES-Assisted Processing
Source: Molecules. 2026 Jan 28;31(3):451. doi: 10.3390/molecules31030451 (PMC12899745; doi:10.3390/molecules31030451)
Supplement: Supplementary file 1 [file molecules-31-00451-s001.zip › molecules-4041187-supplementary.pdf]

Supplementary materials

Valorisation of Barley Straw for Sustainable Nanocellulose Production via Subcritical Alkaline Hydrolysis and HDES-Assisted Processing

Dileswar Pradhan [1,2,3], Swarna Jaiswal [1,2,3], Brijesh K. Tiwari [4] and Amit K. Jaiswal [1,2,3] *

[1] Centre for Sustainable Packaging and Bioproducts Research, Technological University Dublin—City Campus, Central Quad, Grangegorman, D07 ADY7 Dublin, Ireland; dileswar.pradhan@tudublin.ie (D.P.); swarna.jaiswal@tudublin.ie (S.J.)

[2] School of Food Science and Environmental Health, College of Sciences and Health, Technological University Dublin—City Campus, Central Quad, Grangegorman, D07 ADY7 Dublin, Ireland

[3] Sustainability and Health Research Hub (SHRH), Technological University Dublin—City Campus, Central Quad, Grangegorman, D07 ADY7 Dublin, Ireland

[4] Teagasc Food Research Centre, Ashtown, D15 DY05 Dublin, Ireland; brijesh.tiwari@teagasc.ie

* Correspondence: amit.jaiswal@tudublin.ie

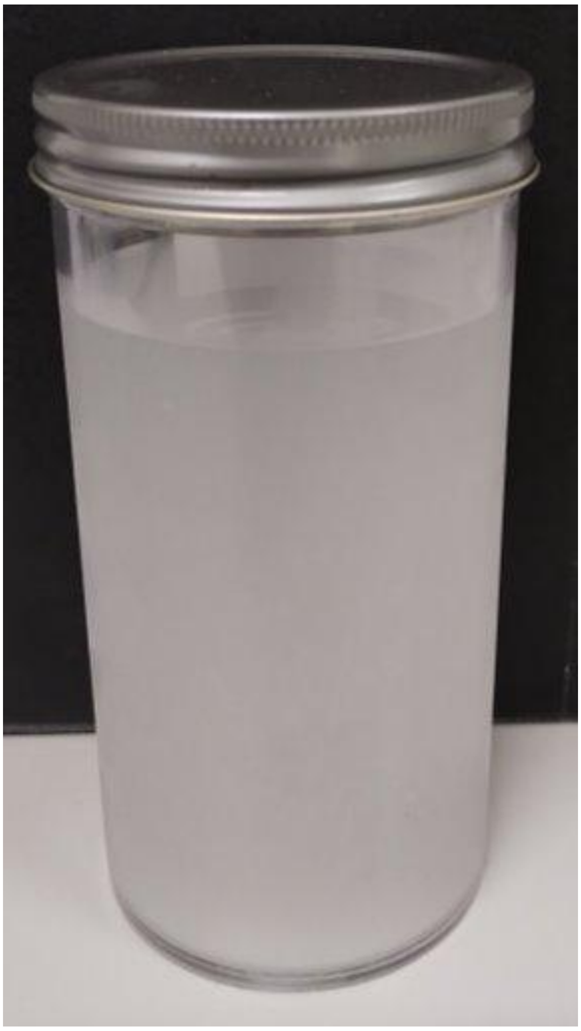

Figure S1. Image of the NC-BTW-3 nanocellulose sample.
